# The clinicopathologic association of c-MET overexpression in Iranian gastric carcinomas; an immunohistochemical study of tissue microarrays

**DOI:** 10.1186/1746-1596-7-57

**Published:** 2012-05-28

**Authors:** Kambiz Sotoudeh, Forough Hashemi, Zahra Madjd, Alireza Sadeghipour, Saadat Molanaei, Elham Kalantary

**Affiliations:** 1 Department of pathology, Tehran University of Medical Sciences, Tehran, Iran; 2 Oncopathology Research Center, Tehran University of Medical Sciences, Tehran, Iran; 3 Department of pathology, Milad Hospital, Tehran, Iran; 4 Department of pathology and Oncopathology Research Center, Tehran University of Medical Sciences, Hemmat highway, Tehran, 1449614531, Iran

**Keywords:** Gastric carcinoma, c-MET, Tissue microarray

## Abstract

**Background:**

c-MET is an oncogene protein that plays important role in gastric carcinogenesis and has been introduced as a prognostic marker and potential therapeutic target. The aim of this study was to evaluate the frequency of c-MET overexpression and its relationship with clinicopathological variables in gastric cancer of Iranian population using tissue microarray.

**Methods:**

In a cross sectional study, representative paraffin blocks of 130 patients with gastric carcinoma treated by curative gastrectomy during a 2 years period of 2008–2009 in two university hospitals in Tehran-Iran were collected in tissue microarray and c-MET expression was studied by immunohistochemical staining.

**Results:**

Finally 124 cases were evaluated, constituted of 99 male and 25 female with the average age of 61.5 years. In 71% (88/124) of tumors, c-MET high expression was found. c-MET high expression was more associated with intestinal than diffuse tumor type (P = 0.04), deeper tumor invasion, pT3 and pT4 versus pT1 and pT2 (P = 0.014), neural invasion (P = 0.002) and advanced TNM staging, stage 3 and 4 versus stage 1 and2 (P = 0.044). The c-MET high expression was not associated with age, sex, tumor location, differentiation grade and distant metastasis, but relative associations with lymph node metastasis (P = 0.065) and vascular invasion (P = 0.078) were observed.

**Conclusions:**

c-MET oncogene protein was frequently overexpressed in Iranian gastric carcinomas and it was related to clinicopathological characteristics such as tumor type, depth of invasion, neural invasion and TNM staging. It can also support the idea that c-MET is a potential marker for target therapy in Iranian gastric cancer.

**Virtual slides:**

The virtual slide(s) for this article can be found here: http://www.diagnosticpathology.diagnomx.eu/vs/9744598757151429

## Introduction

Gastric carcinoma is currently the second leading cause of cancer death in the world. Globally its incidence and mortality declined through the past decades; however it remains the fourth most common cancer in the world. It is estimated that close to one million new cases are occur each year in the world and more than 700.000 deaths are directly related to this problem[1,2].

In Iran, gastric cancer is the second most common and accounts for 10% of all cancers and is the first cause of cancer related mortality in both sexes [3,4]. More than 7300 new cases are occurred annually which mostly presented in stage III and IV and half of them died before the first year of diagnosis. The overall 5-year survival rate of gastric cancer in Iran was 12.8% which is dramatically lower than Japan and developed western countries [4,5].

Surgical approach with or without chemotherapy and/or radiotherapy are the most common modes of therapy. Succumbing to gastric cancer is usually due to local recurrence and distant metastasis, and long term survival after distant metastasis is very low [6].

During the past decades, genetic and molecular information about the mechanisms of gastric carcinogenesis are remarkably improved [7-9]. It is widely accepted that gastric carcinogenesis is a multistage processes, due to interaction between predisposing factors (e.g. *H. Pylori* infection), as well as genetic and epigenetic abnormalities (including activation of oncogenes and/or inactivation of suppressor genes), resulting in uncontrolled cellular growth and dissemination.

Recent studies have shown that alterations in oncogenes encoding tyrosine kinase receptors, play important role in the pathogenesis of gastric carcinoma [10].

c-MET (or MET) is one of the tyrosine kinase receptors' family, encoding a receptor for Hepatocyte Growth Factor (HGF). Activation of c-MET by HGF and its signaling pathways is pivotal for cellular morphogenesis, regeneration, proliferation, migration, angiogenesis and invasion [11]. c-MET is expressed in a variety of normal epithelial and endothelial cells and mediated the biological activities of HGF [12].

Overexpression and amplification of c-MET have been demonstrated in many tumors, including colorectal, thyroid, renal cell, ovary, breast, pancreas, prostate, liver, and melanoma and in gastric carcinoma [13-16].

c-MET overexpression and amplification have been reported in 18-82% of gastric carcinoma which studied by immunofluorescence[12], immunohistochemistry (IHC) [17-27], reverse transcription polymerase chain reaction (RT-PCR) [17,28], Northern blot analysis [12] and Southern blot analysis [19].

In most studies, overexpression of c-MET has been correlated with poor prognosis and it has been regarded as a negative prognostic factor [22,26-29]. Furthermore c-MET overexpression has been strongly associated with local invasiveness and distant metastasis[17], but in few studies the results been discrepant [20,21].

This study was undertaken to examine immunohistochemical expression of c-MET and its clinicopathological association in gastric carcinoma, by using tissue microarray (TMA) technology.

## Methods

In this cross sectional clinicopathological study, gastric cancer patients treated by surgery in two university hospitals (Firoozgar and Rasul) in Tehran-Iran, during a 2 years period of 2008 and 2009 were selected. The inclusion criteria were: proved diagnosis of primary gastric adenocarcinoma and no history of neoadjuvant therapy. The exclusion criteria were lack of suitable block for TMA and insufficient medical records. Eligible specimens were selected from the pathology laboratory files in two hospitals and corresponding medical records were reviewed after institutional research ethics’ committee agreement was obtained.

The pathological features of tumors including location, size, grade of differentiation, tumor classification according to Lauren classification [30], TNM staging according to American Joint Committee on Cancer/International Union against Cancer (UICC) [31], neural and vascular invasions and type of surgery were recorded.

### Tissue microarray construction

The tissue microarrays (TMA) were constructed as described previously [32]. In each case, 5-μm H & E slides were used to identify and mark out representative areas of tumor tissue. From each corresponding paraffin- embedded block, three representative tumor regions were selected. Microarray samples with a diameter of 0.6 mm were punched from selected regions of each “donor” block and precisely arrayed into a new recipient paraffin block using Tissue Arrayer Minicore (ALPHELYS, Plaisir, France). The cores spaced 0.8 mm apart and in each recipient TMA block, 50 ± 5 cores were inserted.

### Immunohistochemistry

Immunohistochemical staining was performed as described previously[33]. On 4 μm tissue sections, mouse monoclonal anti human c-MET (Novocastra- United Kingdom) against external domain of beta chain was used as primary antibody. Briefly, after deparaffinization, endogenous peroxidase activity was inhibited by hydrogen peroxide. Antigens were retrieved by autoclaving and then incubated with primary antibody with an optimal dilution of 1/30. Antigens were visualized using Envision system (DAKO, Denmark) and diaminobenzidine (DAB) (DAKO, Denmark). Finally TMA sections were counterstained with hematoxylin. For processing of the negative control slide, primary antibody was not included. Human prostate tissue was used as positive control for c- MET antibody.

### Evaluation of immunohistochemical staining

The cores with more than 10% tumoral tissue were considered eligible. Within each tissue spot (or core) the most representative tumor region was evaluated and scored by two pathologists that were blind to patients’ data. In case of cores with discrepant scores, cores were re-examined by both pathologists to achieve a consensus score. The membranous and cytoplasmic immunoreactivity in tumoral cells was arbitrarily and semi quantitatively graded by considering the intensity of staining as follow: 0 = negative; 1 = weak (cream); 2 = moderate (light brown); and 3 = strong (brown and dark brown). Cases with scores of 3 were regarded as “high expression” whereas cases with scores of 0–2 were designated as “low expression”.

### Statistical analysis

On completion of collection, data were coded and entered twice into computer files to verify accuracy. Analysis was carried out by SPSS software for windows, version 16.0 (Chicago-IL-USA). Quantitative data are presented as means ± standard deviation. Chi-square test, Fisher exact test and *t*-test were used and P- value less than 0.05 considered significant.

## Results

One hundred and thirty paraffin blocks from 130 gastric cancer patients were collected. Six cases missed in TMA processing and were excluded. Finally 124 cases were analyzed, containing 99 males and 25 females, with the mean age of 61.5 ± 13.1 (range 30–100) years.

### C-MET staining report

**Figure 1:**
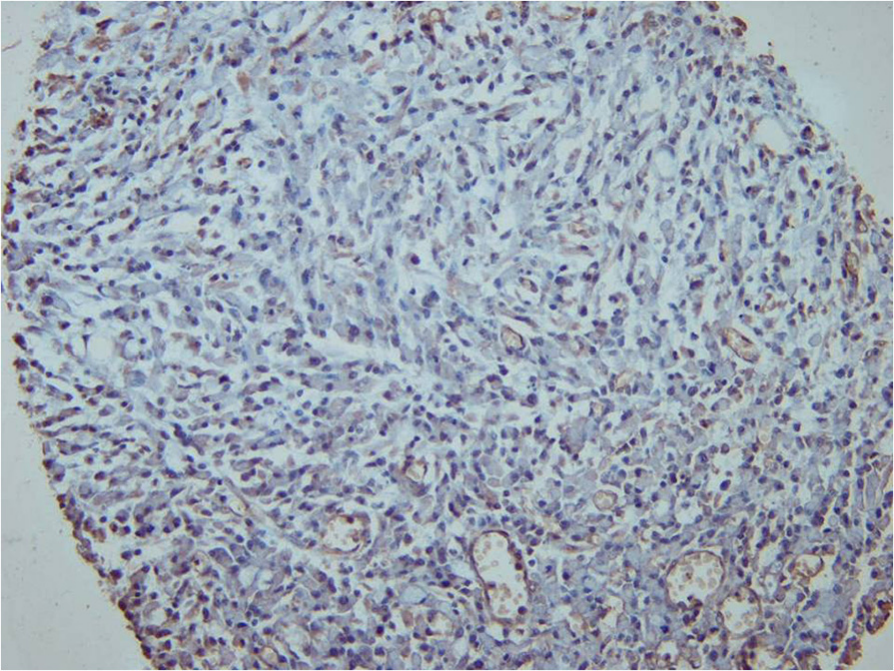
Immunohistochemical staining using c-MET antibody showing weak (+1) cytoplasmic reaction in gastric cancer cells (c-MET, x200).

**Figure 2:**
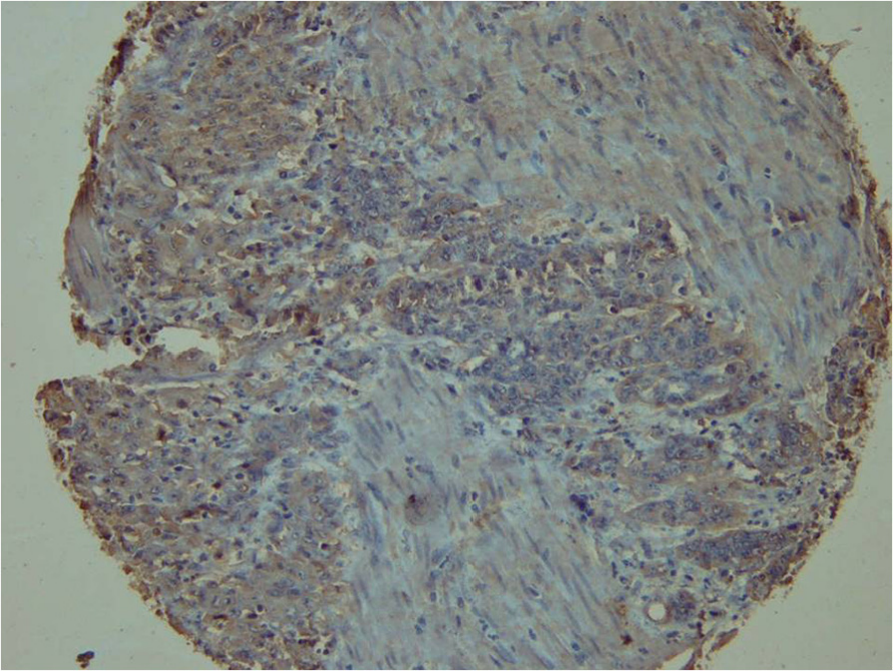
Immunohistochemical staining using c-MET antibody showing moderate (+2) cytoplasmic reaction in gastric cancer cells (c-MET, x200).

**Figure 3:**
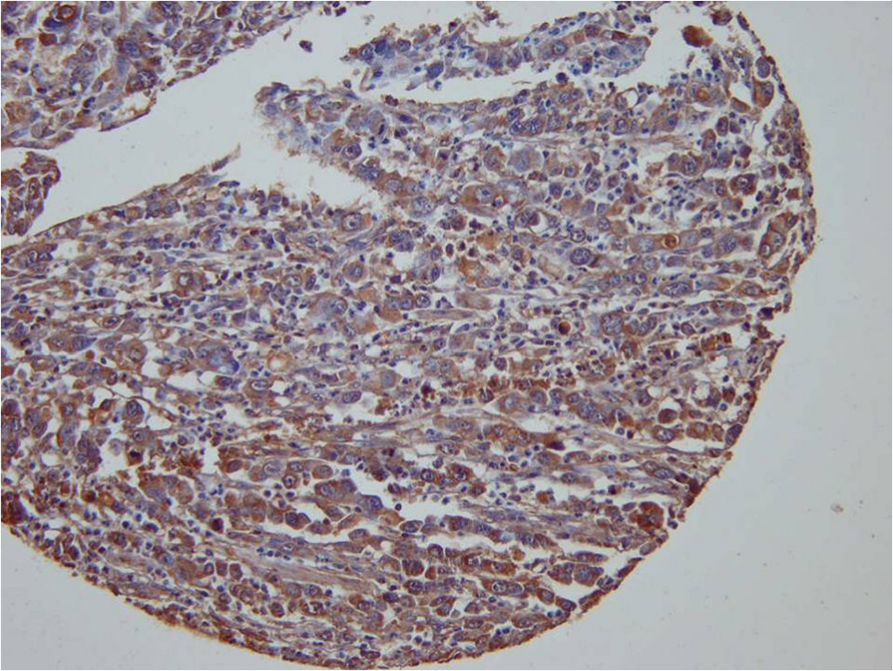
Immunohistochemical staining using c-MET antibody showing strong (+3) and diffuse cytoplasmic reaction in gastric cancer cells (c-MET, x200).

c-MET expression was localized in both cytoplasm and membrane of tumoral cells (Figures 1**,**2 and 3). Occasionally nuclear staining was also seen in tumoral cells. Non tumoral epithelial cells were also show weak to intermediate reactivity. In some cases, stroma was also stained. In most of the tumors, uniform pattern of staining in epithelial tumoral cells was observed. Our criterion for "high expression" of c-MET was strong (3+) staining of cytoplasm and membrane in tumoral cells, which was diagnosed in 88 cases (71%). In high expression of c-MET, there was not possible to differentiate membranous from cytoplasmic staining.

"High expression" of c-MET was more in intestinal type than diffuse type tumors, 77% versus 52%, respectively (P = 0.04). It was also significantly more expressed in deeply invaded tumors, 80% in pT3 and pT4 versus 58% in pT1 and pT2 (P = 0.014). Gastric tumors with neural invasion significantly show high expression of c-MET (83%) compared to those without neural invasion (53%) (P = 0.002). c-MET high expression was also significantly higher in stage 3 and 4 (80%) compared to stage 1 and 2 (44%) of gastric cancer (P = 0.044). In assessment of lymph node metastasis and vascular invasion, there were a relative association (0.05 < P < 0.1) between c-MET expression and both of them. There was no statistical difference between c-MET expression and age, sex, tumor differentiation, tumor location, tumor greatest dimension, distant metastasis and vascular invasion. The relationship between clinical and pathological features and c-MET expression in gastric cancers are summarized in Table 1.

**Table 1:** Relationship between clinicopathological features and c-MET expression in gastric cancers (n = 124)

**Subjects variables**	**c-MET expression**		**P-value**
	**High**	**Low**	
	**(n = 88)**	**(n = 36)**	
**Age (year)**	62.3 ± 12.6	59.5 ±14.4	NS*
**Sex**			
Male	72	27	NS
Female	16	9	
**Tumor location**			
Cardia (upper third)	22	8	NS
Body (middle third)	29	13	
Antrum (lower third)	30	11	
Diffuse	7	4	
**Tumor greatest dimension (cm)**	5.9 ± 3.1	5.7 ± 3.8	NS
**Tumor differentiation**			
Well	11	2	NS
Moderate	32	11	
Poor	45	23	
**Tumor type**			
Intestinal	70	21	0.04
Diffuse	14	13	
Mixed	4	2	
**Depth of invasion**			
pT1	2	5	0.014
pT2	29	17	
pT3	54	13	
pT4	3	1	
**Lymph node metastasis**			
pN0	14	11	0.065
pN1, 2, 3	74	25	
**Distant metastasis**			
Present	9	2	NS
Absent	79	34	
**Tumor stage (TNM)**			
I	9	9	0.044
II	17	11	
III	48	13	
IV	14	3	
**Vascular invasion**			
Present	56	16	0.078
Absent	31	18	
Unknown	1	2	
**Neural invasion**			
Present	60	12	0.002
Absent	23	20	
Unknown	5	4	

NS = not significant.

## Discussion and conclusions

In Iran, gastric cancer is the second most common cancer and the leading cause of cancer death and is an important health problem because of high risk areas in north and northwestern regions [3]. However about 80% of the patients were presented in advanced stages and did not gain any survival benefit from conventional therapy [3-5]. Therefore new strategy based on the screening of high risk population and achieving new prognostic factors to predict the behavior of gastric tumor as well as personalized target therapy are required.

The era of target therapy has started in 1990 by Tamoxifen and until now more than 38 agents have been introduced for various cancers, among them anti-HER2/neu and anti c-kit therapy are the known ones. While c-kit expression in gastric carcinoma is very limited, Her2-neu expressed in less than 20% of them [34,35], and efforts for finding other molecular biomarker are currently in development.

c-MET is an oncogene that has gained attention as a prognostic marker, and an indicator of metastasis and poor prognosis as well as a good target for therapeutic inhibition [16]. Until now many new anti-c-MET drugs have been invented and most are in preclinical and clinical testing [34,36].

Previous studies have been shown c-MET overexpression in selected patients indicates that certain patients may be sensitive to targeted therapy [37]. Moreover it has been confirmed that this protein overexpression is significantly associated with high level of c-MET mRNA and gene amplification [19,28].

In the current study, c-MET overexpression was observed in 71% of gastric cancers and showed significant statistical relationships with tumor type and depth of tumor invasion as well as neural invasion and TNM staging. It also showed a relative relationship with vascular invasion and lymph node metastasis.

As far as we know this is the first time that c-MET oncogene is evaluated by Tissue microarray in Iran. Tissue microarray technology has many advantages including: facilitate the staining and the interpretation and reduces the intra – and inter observer variation of IHC interpretation and particularly saving the cost. We used a semi automatic instrument but a new simple method is also introduced by using mechanical pencil tips with much more low costs [38].

There were few studies using TMA to evaluate c-MET by IHC in gastric cancer [18,21,39] and all were performed in China.

Tang et al. studied c-MET expression in normal gastric mucosa, intestinal metaplasia and gastric cancer and found c-MET overexpression in 68.8% of 232 gastric cancers. Its expression was significantly higher than intestinal metaplasia. They did not find any relationship between c-MET expression in gastric cancer with tumor stage, grade of differentiation or tumor type [21].

In study by Zhao et al., c-MET expression was identified in cases of gastric carcinoma and matched normal gastric mucosa, as well as cases with chronic atrophic gastritis, intestinal metaplasia and dysplasia. c-MET overexpression was found in about 66% of gastric carcinoma which was significantly higher than normal mucosa, chronic gastritis, metaplasia and dysplasia. Its overexpression was associated with tumor type (more in intestinal than in diffuse type), grade of differentiation and lymph node metastasis [39].

Another study was also recently performed in China by Li et al., and they reported c-MET expression in 82.4% of 114 gastric cancer. They found significant association between c-MET expression and advanced clinical stage, lymph node metastasis and poor prognosis [18].

In contrast to Tang et al. [21], our findings are in agreement with Li et al. [18] and Zhao et al. [39] studies and in continuation of earlier studies that reported c-MET association with poor prognosis and local invasiveness.

In present study we did not assess survival rate because of the short term of study and limitation of patients' data, differences in patients' management and lack of regular patients' follow up.

Another difference between the current study and three above mentioned studies is in interpretation of c-MET reactivity. Zhao et al. and Li et al. used double score or product score (multiply intensity by area) with different criteria and cut offs, and Tang et al. used only the area of reactivity with the cut off of 10%; however we used only "intensity" of reaction.

Traditionally, some authors have used double score or H score (multiply intensity by area) in evaluation of IHC staining, but we more agree with Tolgay et al. [40] that due to the minute area (0.6 mm diameter) of the spots in TMA and homogeneity in their reactions, ignoring "area" is more appropriate in this setting.

In other studies using whole slide evaluation (without using TMA), a spectrum of criteria have been used to defining a positive reaction, but many used "area" with various cut offs of 5% to 30% [17,19,22,28,29], and few used a product score (intensity × area) [41].

It does not need to emphasis that with these various arbitrary criteria, either in TMA or in whole slide assessment, we will require a general consensus in regard to evaluation of c-MET expression in gastric cancer. It would not far that anti c-MET drugs become a part of cancer management and defining eligible patient would be our duty [37]. In fact standardization of various types of antibodies used against c-MET would be another part of this work. Until now, antibodies commonly used are against extracellular alpha and beta subunits as well as against intra cytoplasmic c-terminal. It is not clarified that regardless of the name of producers, which type of antibody is more sensitive or specific.

Some studies reported more c-MET expression in diffuse tumors than in intestinal type [41-43]. In contrast to these results, Drebber et al. and Zhao et al. [29] reported more c-MET expression in intestinal tumors, and in agreement with them we also found a significant expression of c-MET in intestinal tumors than in diffuse.

TNM staging system according to The American Joint Committee on Cancer (AJCC)/International Union against Cancer (UICC) produced the most reliable system for predicting the survival of patients. Furthermore lymphatic and vascular invasion were considered as poor prognostic indicators [8,31,44,45].

In the present study, c-MET expression was significantly related to depth of tumor invasion (pT), and TNM staging and at least implied as a marker of local invasiveness. As the depth of invasion and perforation of serosa were introduced as independent prognostic factors [44], thus c-MET expression could be regarded as a potential prognostic factor. This fact has been also affirmed by other studies [17,19,23,28,46].

c-MET expression in present study showed no relationship to tumor differentiation which was in agreement with Tang et al. results [21].

Several studies reported that c-MET expression had a remarkable relationship with lymph node metastasis [18,19,22,26,27,39] while in our study there was a relative relationship (P = 0.065) between these two variables. One logical reason is that the average number of separated lymph nodes in our cases was 11, which was under the standard number of at least 15 per each gastrectomy sample [8], and another reason was related to type of surgery. The method of extended lymphadenectomy in gastrectomy which widely used in Japan and other countries did not advocate in Iran. For example in one study using extended lymphadenectomy an average of 39 lymph nodes was resected for each patient [29].

In the present study in agreement with previous studies, association of c-MET overexpression and advanced stage of gastric carcinoma was seen [12,13,17,19,28], while some authors reported discrepant results [20,21,29] .

Nakajima et al. [19] found c-MET as a negative independent prognostic factor and this finding was verified by others [26-28] .

In the present study we did not find any association between distant metastasis and c-MET expression, which was in agreement with Huang et al. [28] study and in contrast to Amemiya et al. [17] that showed all gastric cancers with distant metastasis overexpressed c-MET, but due to limited number of metastatic cases in our study, their findings cannot be ruled out.

In conclusion c-MET oncogene protein was frequently overexpressed in Iranian gastric carcinomas and it was related to clinicopathological characteristics such as tumor type, depth of invasion, neural invasion and TNM staging. It can also support the idea that c-MET is a potential marker for target therapy in Iranian gastric cancer.

## Competing interest

Authors declare no conflict of interest.

## Authors' contributions

KS designed the study and performed case selection, immunohistochemical analysis, writing and editing the manuscript, data analysis and interpretation. FH and ZM participated in immunohistochemical analysis and interpretation, writing and editing the manuscript. AS and SM participated in case selection, immunohistochemical interpretation. EK carried out immunohistochemistry and tissue microarrays. All authors read and approved the final manuscript.
